# Supplementary material for: In Silico and In Vitro Studies of Benzothiazole-Isothioureas Derivatives as a Multitarget Compound for Alzheimer’s Disease
Source: Int J Mol Sci. 2022 Oct 26;23(21):12945. doi: 10.3390/ijms232112945 (PMC9658106; doi:10.3390/ijms232112945)
Supplement: Supplementary file 1 [file ijms-23-12945-s001.zip › ijms-1937122-supplementary.pdf]

International Journal of *Molecular Sciences*

MDPI

Supplementary Material

In silico and in vitro studies of benzothiazole-isothioureas derivatives as a multitarget compound for the Alzheimer's disease.

Martha Cecilia Rosales Hernández*[1], Leticia Guadalupe Fragoso Morales[1], José Correa Basurto[2], Marycruz Olvera Valdez[1,4], Efrén Venancio García Báez[3], Dania Guadalupe Román Vázquez[1], Ana Paola Anaya García[1] and Alejandro Cruz*[3]

Table S1. ΔG values and amino acids residues of $A\beta_{1-42}$ in α-helix conformation that did interaction with the benzothiazole-isothioureas.

Focused Docking $A\beta_{1-42}$ (α)		
Compound	ΔG (kcal/mol)	Residues
1	-3.96	K16, L17, H13, H14, Q15, V18, A21, E22
2	-4.07	F19, Q15, H14, E11, V18, D23, E22, N27
3	-6.02	F20, F19, Q15, V12, H14, E11, D7, V18, E22, D23, N27
4	-4.33	F20, F19, Q15, V12, H14, E11, Y10, V18, E22,
5	-4.41	K16, L17, H13, H14, Q15, V18, A21, E22
6	-6.02	F20, F19, Q15, V12, H14, E11, D7, V18, E22, D23, N27
7	-4.99	Phe -20, F19, Q15, V12, H14, E11, Y10, V18, E22
8	-4.45	N27, A30, L34, M35, I31, A42, I41, V40, V39
9	-5.23	F19, Q15, V12, H14, E11, Y10, V18, E22, D23,
10	-8.17	F20, F19, Q15, V18, E22, A21, D23, V24, K28, N27, S26
11	-4.91	V40, I41, A42, M35, I32, I31, K28, N27, A30, G29, L34
11M	-6.77	N27, D23, F19, L17, K16, V12, H13, Q15, V18, E22, S26
12	-5.11	N27, D23, F20, F19, V18, Q15, H14, V12, E11, Y10, E22
13	-4.87	N27, D23, E22, F19, V18, K16, Q15, H14, H13, V12, E11, Y10
14	-5	A42, I41, V39, V40, D23, K28, N27, I31, A30, I32, M35, L34
15	-5.81	A42, I41, V40, M35, I31, K28, A30, I32, L34
16	-4.69	F20, L17, K16, F19, Q15
17	-7.12	A42, V40, I41, M35, L34, I31, A30, N27, K28, D23, F19, F20
18	-6.37	N27, K28, D23, V24, A21, F20, E22, F19, Q15, V18, H14, E11, V12, Y10
19	-5.75	V39, A42, V40, I41, M35, I31, A30, N27, K28, D23, F19, V24, F20
20	-6.42	N27, D23, F19, E22, F20, V18, Q15, V12, E11, H14
21	-5.22	N27, K28, D23, V24, A21, F20, E22, F19, Q15, V18, H14, E11
22	-5.33	N27, D23, S26, F20, E22, F19, V18, Q15, V12, D7, E11, H14, Y10
23	-6.87	V39, A42, V40, I41, M35, L34, I31, A30, N27, K28, D23, V24, F20

Table S2. ΔG values and amino acids residues of $A\beta_{1-42}$ in β-sheet conformation that did interaction with the benzothiazole-isothioureas.

Focused Docking $A\beta_{1-42}$		
Compound	ΔG (kcal/mol)	Residue
1	-3.62	M35, V36, A21, E22, D23, L34, G25, V24
2	-3.85	M35, V36, A21, E22, D23, L34, G25, V24
3	-4.93	G37, G38, F19, F20, A21, V36, E22
4	-4.06	G37, G38, F19, F20, A21, V36, E22
5	-4.18	G37, G38, F19, F20, A21, V36, E22
6	-4.99	M35, V36, A21, E22, D23, L34, G25, V24
7	-4.53	M35, V36, A21, E22, D23, L34, V24, I32
8	-3.94	G37, V36, M35, L34, G33, A21, E22, D23
9	-4.93	M35, V36, L34, I32, A21, E22, D23, V24, G25

10	-6.5	V18, F19, F20, A21, E22, D23, V24
11	-4.49	G37, V36, M35, L34, G33, I32, F19, A21, E22, D23
11M	-6.07	M35, V36, L34, G33, I32, A21, E22, D23, V24, G25
12	-4.74	G37, V36, M35, L34, I32, A21, E22, D23, V24, G25
13	-4.75	G37, V36, M35, L34, G33, A21, E22, D23
14	-4.83	G33, L34, D23, V24, G25, I32, I31, A30, G29, K28, N27, S26
15	-4.37	G37, V36, M35, L34, G33, F19, F20, A21, E22, D23, V24, G25
16	-4.72	L34, D23, V24, G25, I32, I31, A30, G29, K28, N27, S26
17	-6.66	L34, I32, F19, F20, A21, E22, D23, V24, G25, N27, S26, G29 K28
18	-5.95	G37, G38, M35, V36, L34, F19, F20, A21, E22, D23
19	-5.95	G37, V36, M35, L34, F19, A21, E22, D23, V24, G25
20	-6.26	G37, G38, M35, V36, L34, F19, F20, A21, E22, D23, V24
21	-4.92	V39, G38, G37, V36, M35, L34, F19, F20, A21, E22, D23
22	-5.41	G37, V36, M35, L34, F19, F20, A21, E22, D23, V24, G25
23	-6.17	G37, V36, M35, L34, G33, I32, A21, E22, D23, G25, V24

Table S3. ΔG values and amino acids residues of A$\beta_{1\text{-}42}$ in Random Coil conformation that did interaction with the benzothiazole-isothioureas.

Focused Docking in RC.		
Compound	ΔG (kcal/mol)	Residue
1	-3.55	G9, Hsd-13, Q15, Hsd-14, F20, V18, F19, D23
2	-3.62	Hsd-13, Q15, Hsd-14, V18, F19, D23
3	-4.73	I32, I31, G33, A30, G29, A21, F20, K28, G25, S26, N27
4	-3.21	Q15, Hsd-14, V18, F19, D23
5	-3.52	Q15, Hsd-14, V18, F19, D23
6	-4.72	I32, I31, G33, A30, G29, A21, F20, K28, G25, S26, N27
7	-4.22	Hsd-13, Q15, Hsd-14, V18, F19, D23
8	-3.57	I32, I31, A30, A21, F20, S26, G25, G29, K28, N27
9	-3.72	I32, I31, A30, A21, F20, E22, G25, G29, K28, S26, N27
10	-6.02	Hsd-14, V18, F19, D23, V24, S26
11	-3.9	I32, I31, A30, A21, E22, G25, K28, G29, S26, N27
11M	-5.96	L34, F19, D23, V24, G25, K28, S26, N27
12	-4	I32, I31, G33, A30, G29, A21, F20, K28, G25, S26, N27
13	-4.24	I31, L17, V18, E22, A21, F20, A30, G29, K28, G25, S26, N27
14	-4.76	I32, I31, G33, A30, G29, A21, F20, K28, G25, E22, S26, N27
15	-5.17	E22, D23, G25, V24, S26, N27, K28
16	-5.19	F19, D23, V24, G25, K28, S26, N27
17	-6.05	Hsd-13, L17, V18, E22, A21, I31, A30, G25, S26, Ans-27, K28, G29
18	-4.85	Hsd-13, L17, V18, A21, E22, D23, G25, S26
19	-5.02	Hsd-13, L17, V18, F20, A21, E22, I31, A30, G25, S26, N27, K28, G29
20	-5.27	L17, V18, A21, E22, D23, G25, S26
21	-3.19	Hsd-13, Hsd-14, F19, V18, E22, D23
22	-2.61	Hsd-14, L17, V18, F19, A21, E22, Aps-23, G25, S26
23	-5.01	I31, A30, V18, A21, G29, E22, Aps-23, G25, V24, K28, S26

Table S4. ΔG values and amino acids residues of AChE that did interaction with the benzothiazole-isothioureas.

Focused Docking AChE		
Compound	ΔG (kcal/mol)	Residue
1	-5.16	F338, F295, V294, Y341, T83, D74
2	-5.56	F338, F295, V294, Y341, T83, D74
3	-7.48	R296, F295, V294, F338, Y341, D74, W86
4	-5.72	F338, F295, V294, Y341, T83, D74
5	-5.76	G121, G122, T83, F295, V294, F338
6	-7.49	W86, D74, R296, F295, V294, Y341, F338

7	-6.19	F295, V294, T83, G121, G122
8	-5.73	D74, N87, W86, Trh-83
9	-6.42	F295, V294, T83, G121, G122
10	-8.19	F295, V294, Y341, F338, G448, W86, G121, G126
11	-6.27	G122, R296, F295, V294, S293, F338, Y341, G342, D74
11.5	-8.66	G121, G448, H447, F338, T83, D74, W86, N87
12	-6.44	G121, G122, F295, V294, T83
13	-7.01	G120, G121, G122, F295, V294, F338, T83
14	-7.47	F297, R296, F295, V294, F338, Y341, D74
15	-6.08	F295, V294, F338, Y341, L339, T83, D74
16	-5.64	G121, F295, V294, F338, Y341, L339, D74
17	8.59	G126, G120, G121, W286, Y341, D74, W86
18	-7.26	G120, G121, R296, F295, V294, S293, Y341, F338, Trh-83, D74, W86, N87
19	-3.66	G126, G120, G121, G122, F295, V294, G342, Y341, F338, W86
20	-8.03	G121, G122, F295, V294, Y341, F338, T83, D74, W86
21	-7.47	G121, G122, R296, F295, V294, S293, G342, Y341, T83, D74, W86
22	2.62	F297, R296, F295, V294, F338, Y341, T83, W86, D74, G121, S203, G122, A204
23	-6.65	S293, F295, V294, G342, Y341, D74

Table S5. SMILE code of **3f**, **3r** and **3t** compounds

Compounds	SMIS
3f	CSC(\NC1C2CC3CC(C2)CC1C3)=N\c1nc2ccccc2s1
3r	CS\C(=N\c1nc2ccccc2s1)Nc1ccc(cc1)N/C(SC)=N\c1nc2ccccc2s1
3t	CSC(\NCCN1CCN/C1=N\c1nc2ccccc2s1)=N\c1nc2ccccc2s1

Table S6. Lipophilicity properties of **3f**, **3r** and **3t** compounds

**Molecule**	**iLOGP**	**XLOGP3**	**WLOGP**	**MLOGP**	**Silicos-IT Log P**	**Consensus Log P**
**3f**	3.75	5.5	5.06	4.38	4.73	4.68
**3r**	3.96	7.42	7.45	4.48	7.09	6.08
**3t**	4.22	4.84	3.68	3.52	5.58	4.37

Table S7. Water solubility of **3f**, **3r** and **3t** compounds

**Molecule**	**ESOL**				**Ali**				**Silicos-IT**			
	**Log S (ESOL)**	**Solubility (mg/ml)**	**Solubility (mol/l)**	**Class**	**Log S (Ali)**	**Solubility (mg/ml)**	**Solubility (mol/l)**	**Class**	**LogSw (Silicos-IT)**	**Solubility (mg/ml)**	**Solubility (mol/l)**	**Class**
**3f**	-5.54	1.04E-03	2.92E-06	Moderately soluble	-7.17	2.44E-05	6.83E-08	Poorly soluble	-5.18	2.34E-03	6.55E-06	Moderately soluble
**3r**	-7.74	9.53E-06	1.83E-08	Poorly soluble	-11.07	4.48E-09	8.60E-12	Insoluble	-9.66	1.14E-07	2.19E-10	Poorly soluble
**3t**	-5.76	8.20E-04	1.75E-06	Moderately soluble	-7.92	5.56E-06	1.19E-08	Poorly soluble	-7.62	1.12E-05	2.40E-08	Poorly soluble

Table S8. Pharmacokinetics parameters of **3f**, **3r** and **3t** compounds.

Molecule	GI absorption	BBB permeant	Pgp substrate	CYP1A2 inhibitor	CYP2C19 inhibitor	CYP2C9 inhibitor	CYP2D6 inhibitor	CYP3A4 inhibitor	log Kp (cm/s)
**3f**	High	No	Yes	Yes	Yes	Yes	Yes	No	-4.58
**3r**	Low	No	No	No	Yes	Yes	No	Yes	-4.21
**3t**	Low	No	Yes	Yes	Yes	Yes	Yes	Yes	-5.72

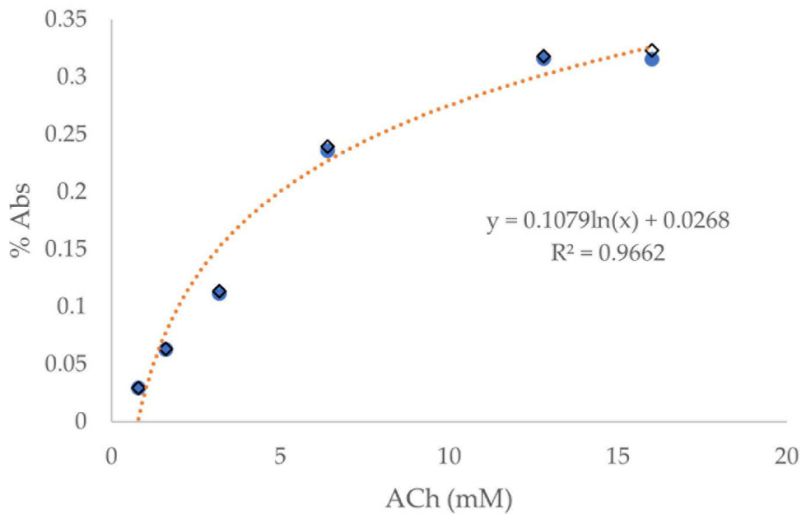

Figure S1. AChE kinetic with and without DMSO 0.4% in PBS at pH 8.

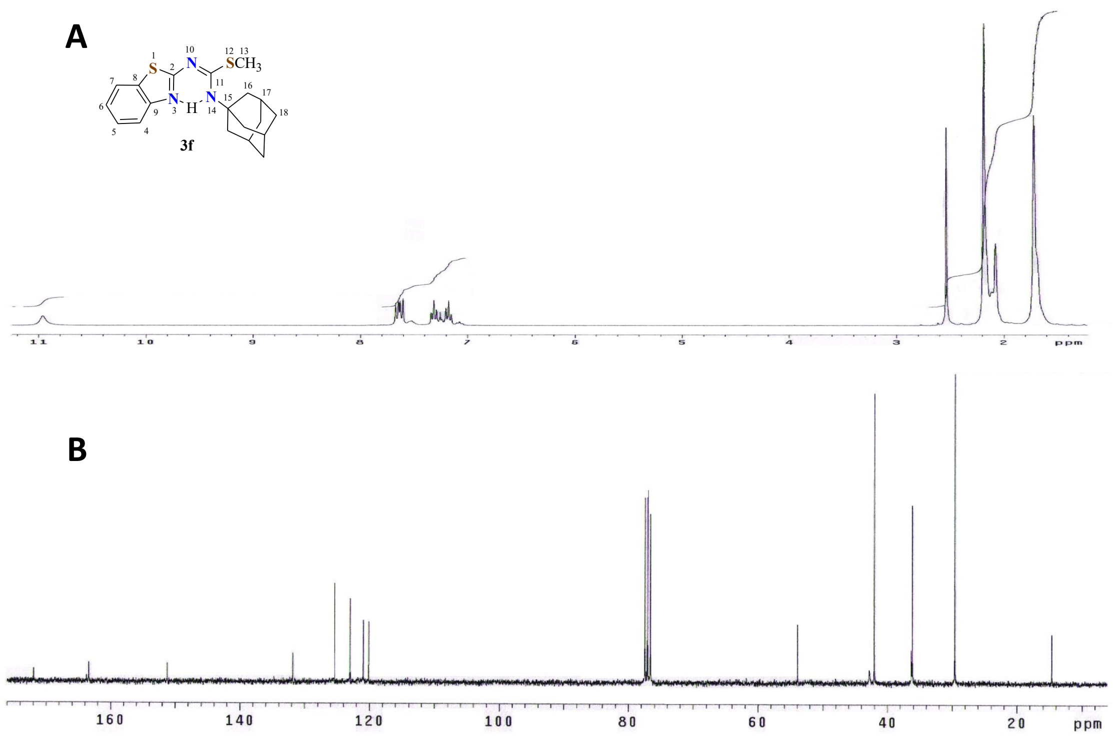

Figure S2. A) 1H and B) 13C NMR spectra of compound **3f**.

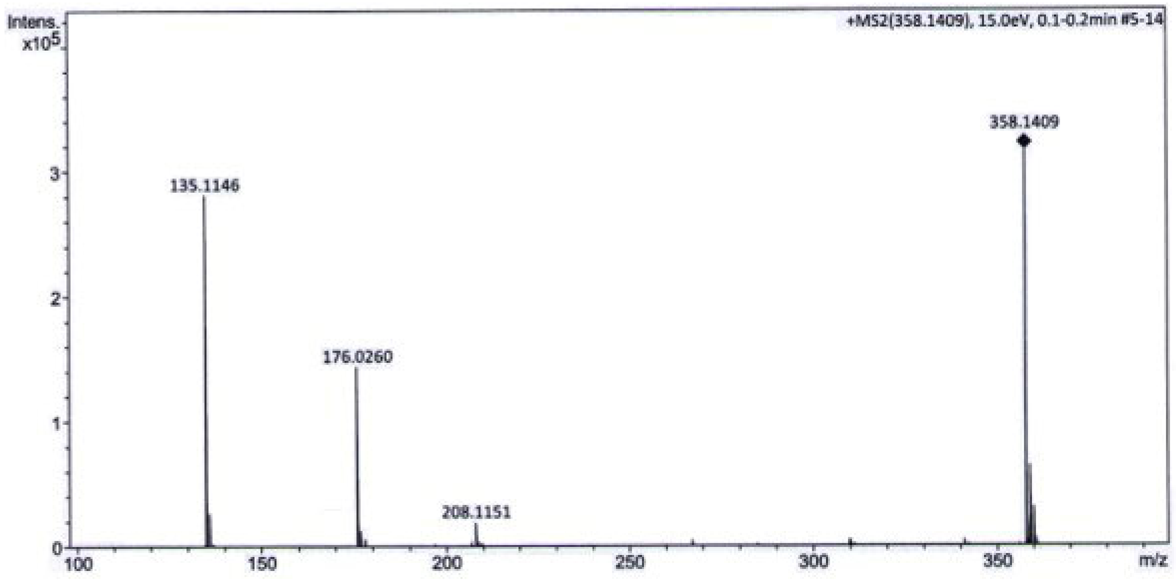

Figure S3. ESImass spectrum of compound **3f**.

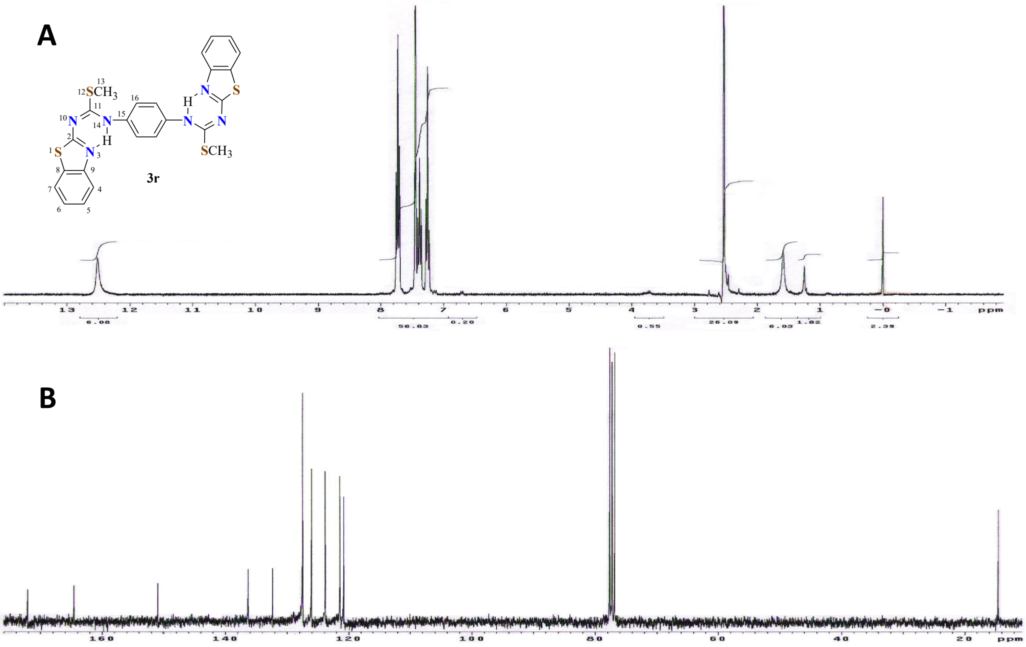

Figure S4. A) 1H and B) 13C NMR spectra of compound **3r**.

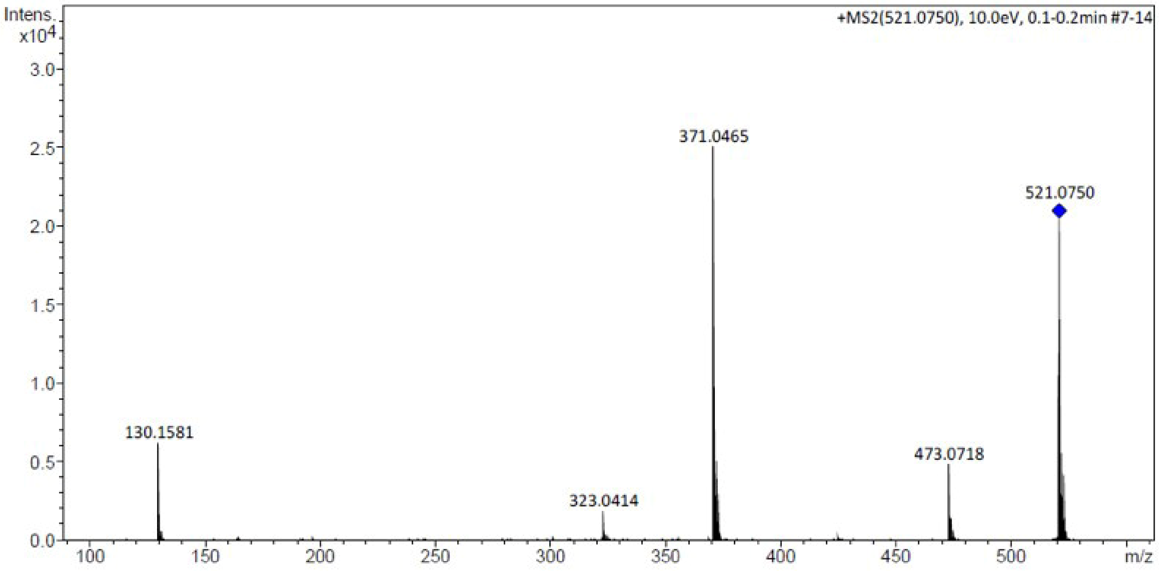

Figure S5. ESImass spectrum of compound **3r**.

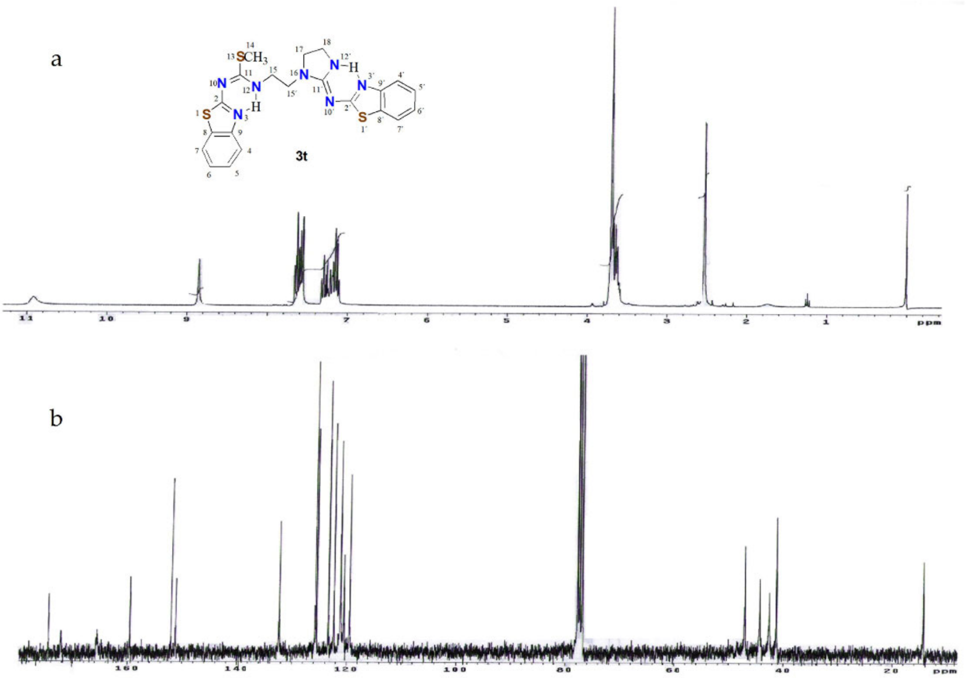

Figure S6. A) 1H and B) 13C NMR spectra of compound **3t**.

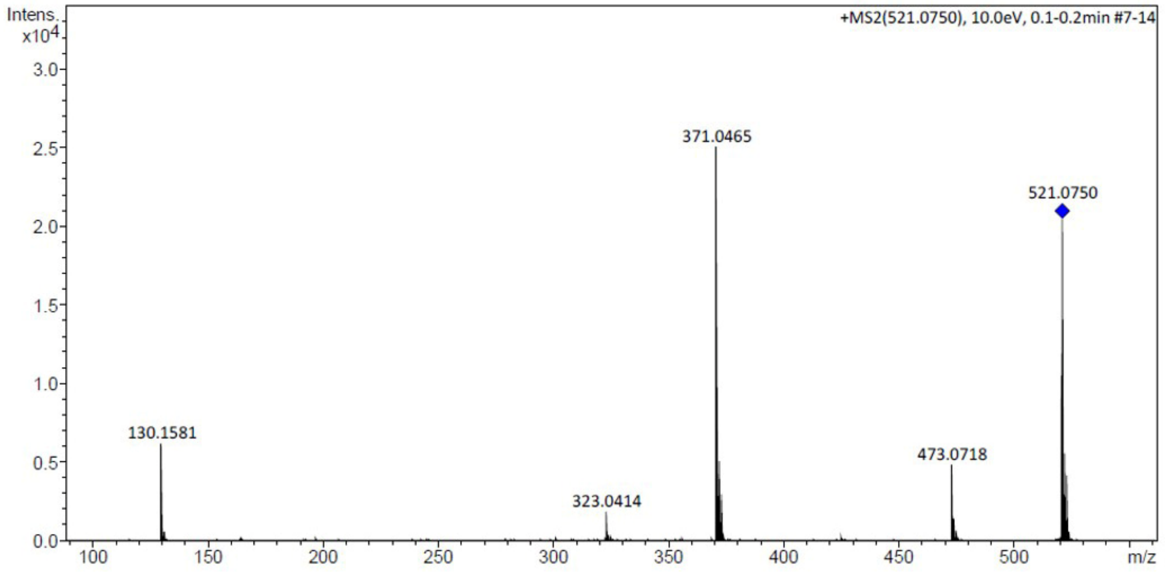

Figure S7. ESImass spectrum of compound **3t.**
